# Cerebral ischemia detection using deep learning techniques

**DOI:** 10.1007/s13755-025-00352-8

**Published:** 2025-05-20

**Authors:** Rafael Pastor-Vargas, Cristina Antón-Munárriz, Juan M. Haut, Antonio Robles-Gómez, Mercedes E. Paoletti, José Alberto Benítez-Andrades

**Affiliations:** 1 Communications and Control Systems, Computer Engineering Science Faculty, UNED, Calle Juan del Rosal 16, 28040 Madrid, Spain; 2 Radiology area, Hospital Universitario de Navarra, Navarra, Spain; 3 School of Technology, University of Extremadura, Cáceres, Extremadura Spain; 4 ALBA Research Group, Department of Electric, Systems and Automatics Engineering, University of Leon, Campus of Vegazana s/n, 24071 León, Castilla y León Spain

**Keywords:** Cerebral ischemia, Computed tomography, Deep learning, Transfer learning, Ictus dataset

## Abstract

Cerebrovascular accident (CVA), commonly known as stroke, stands as a significant contributor to contemporary mortality and morbidity rates, often leading to lasting disabilities. Early identification is crucial in mitigating its impact and reducing mortality. Non-contrast computed tomography (NCCT) remains the primary diagnostic tool in stroke emergencies due to its speed, accessibility, and cost-effectiveness. NCCT enables the exclusion of hemorrhage and directs attention to ischemic causes resulting from arterial flow obstruction. Quantification of NCCT findings employs the Alberta Stroke Program Early Computed Tomography Score (ASPECTS), which evaluates affected brain structures. This study seeks to identify early alterations in NCCT density in patients with stroke symptoms using a binary classifier distinguishing NCCT scans with and without stroke. To achieve this, various well-known deep learning architectures, namely VGG3D, ResNet3D, and DenseNet3D, validated in the ImageNet challenges, are implemented with 3D images covering the entire brain volume. The training results of these networks are presented, wherein diverse parameters are examined for optimal performance. The DenseNet3D network emerges as the most effective model, attaining a training set accuracy of 98% and a test set accuracy of 95%. The aim is to alert medical professionals to potential stroke cases in their early stages based on NCCT findings displaying altered density patterns.

## Introduction

An obstruction of a cerebral artery by a thrombus or embolus can lead to a lack of blood flow in the irrigated territory of the vessel. Consequently, it results in cellular ischemia, leading to cell death if vascular obstruction persists [1]. The ischemia can be reverted in the initial hours if it is detected on time. If not detected in time, this obstruction can produce infarction or neuronal death. Typically, the anterior circulation is the most frequent location of ischemic stroke in the circulation. In particular, internal carotid, anterior and middle cerebral arteries (MCA) are located in this area, being the obstruction of the MCA the more frequent location for the embolus/thrombus. To avoid the irreversible problems discussed above, once the initial symptoms are detected, it is vital to treat intravenous thrombosis within 4–6 h. After this time, hemorrhagic complications can occur, leading to neuronal death [2, 3].

**Fig. 1:**
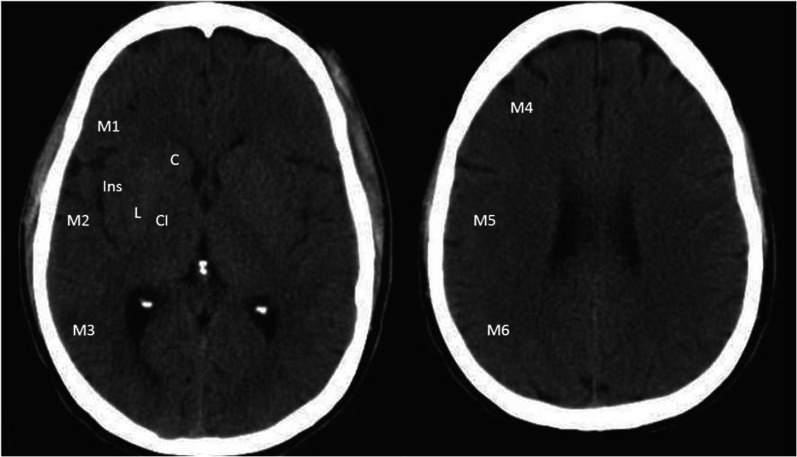
ASPECTS areas: Insula (I), Lenticular (L), Caudate (C), Capsule Internal (CI) and cortical areas: M1, M2, M3, M4, M5 and M6

To assess early signs of middle cerebral artery (MCA) involvement, the Alberta Stroke Program Early Computed Tomography Score (ASPECTS) is utilized. This scoring system ranges from 0 to 10, based on the presence of hypodensity in ten brain regions: the lenticular nucleus, insula, caudate nucleus, internal capsule, and six cortical areas [4]. A score of 10 on the ASPECTS scale indicates no signs of early ischemia. A score below 7 indicates a worse prognosis and an increased risk of hemorrhage as a complication of thrombolytic therapy. Our dataset includes NCCTs with hypodensity in one or more territories, as shown in Fig. 1.

The diagnosis of hypodensities is a challenge that relies heavily on the expertise of the radiologist/physician in using and receiving hypodensities from an NCCT. Hypodensities are very faint, making it difficult to observe the areas where they appear accurately. Since these changes in the hypodensities are not very noticeable, it is necessary to use more advanced techniques such as deep neural networks or deep learning. These networks are characterized by looking for visual patterns in the images they analyze, which are an excellent aid for radiologists/physicians if appropriately used. The neural networks need to be trained to trust in their decisions, and once the correct metrics are established, they can be of great value in detecting the presence of an infraction.

Medical imaging is one of the most widely used areas of neural networks, particularly Convolutional Neural Networks (CNNs). These networks can extract information from the image, such as non-linear features not visible to the human eye. The use of these CNNs and their application in the diagnosis of diseases is booming, especially among researchers in the field of medical informatics. The fields of application are very broad, given the number of existing mechanisms for obtaining images (or equivalent representations) associated with the diagnostic problem.

For the particular case of stroke, 3D images are used. 3D neural networks are an area of development that is being used in contrast to neural networks that use two-dimensional images. The ability to discover in three dimensions makes it possible to obtain patterns that traditional 2D networks cannot learn. However, 3D networks require much more processing power, so it is necessary to use parallel processing technologies and more concerted use of graphics processing units or GPUs. The use of these 3D models has provided specific tools for diagnosis by segmenting and classifying medical images of the specific domain [2].

Other artificial intelligence algorithms, such as supervised machine learning algorithms, are already being used. Applications such as e-ASPECTS use these algorithms that are more limited than neural networks. e-ASPECTS uses the random forest algorithm to compare healthy hemispheres with those affected [5–7]. The segmentation of the images must be done manually, and in this case, it must be performed by an experienced radiologist to have acceptable results. Given the limitations of random forests, this work chooses to use neural networks since CNNs are being used in the area of medical imaging for stroke diagnosis using Computed Tomography (CT) as in [8], [9] and [10]. An extensive review of deep learning algorithms for CT images can be found in [11]. Other works focus on the application of CCNs using MRI (Magnetic Resonance Imaging), like the one presented in [12].

For this paper, the research will not use CT or MRI but rather NCCT images. There are plenty of works on CNNs using CT/MRI images but fewer using NCCT images. Still, given the good results of CNNs with CT images, the research hypothesis is to obtain similar results with NCCT images. This hypothesis can be corroborated by experimental results, primarily through the application of deep learning transfer techniques.

The rest of the paper is organized as follows. Section 2 presents the importance of the use of convolutional neural networks in the area of medical imaging. This section focuses on using these tools to assist physicians/radiologists in their decisions. Section 3 indicates how the data set used for network training has been constructed. This data set does not exist in recent academic literature or the data repositories analyzed. For this reason, the construction procedure is shown for reproduction if necessary. Section 4 details the experiments developed and the data obtained for the defined metrics in order to be used to compare the different results. Section 5 discusses the results and the factors used to decide on the neural network for the decision assistance process. Finally, in Sect. 6, the conclusions obtained and future work are shown.

## Deep learning and medical images

Since its inception in 1943 by McCulloch and Pitt [13], the field of Artificial Neural Networks (ANN) has witnessed a significant transformation, particularly notable in recent years. The seminal work by McCulloch and Pitt introduced a mathematical framework for ANNs, emphasizing the binary nature of neuronal activity and their representation through a threshold function. Neural networks, particularly through the utilization of non-linear activation functions in hidden layers, offer a robust mechanism for modeling complex, non-linear relationships inherent in the training data, a task that is challenging for traditional algorithms. Among the various architectures developed, CNNs, cited in [14], have gained prominence in tasks involving image data, such as in the field of medical imaging. The rapid advancements in deep learning, aligned with breakthroughs in biological and medical imaging technologies, have led to an unprecedented ability to generate, collect, and analyze voluminous datasets of medical images, thereby enhancing the capability of CNNs in feature detection and pattern analysis.

### CCN models

Numerous established applications in the fields of medicine and biology have successfully utilized CNNs for tasks such as image classification and segmentation, similar to the approach we have taken. These networks are instrumental across a diverse array of challenges including, but not limited to, cancer detection, continual disease monitoring, the generation of customized treatment plans, and accurate disease diagnosis. Furthermore, CNNs have demonstrated versatility in handling a variety of data sources including but not limited to X-rays, computed tomography (CT) scans, magnetic resonance imaging (MRI), retinography, pathological anatomy slides, and even sequences from the human genome, as cited in [15].

The subsequent examples represent merely a fraction of the applications employing CNNs in medical imaging, showcasing the breadth of ongoing research in this expansive field. These applications illustrate the potential of CNNs to classify a multitude of diseases, employ various imaging modalities, and utilize a diverse range of convolutional network architectures. In the realms of lung nodule classification, detection, and segmentation using CT scans, various deep learning approaches have been employed. These include 2D and 3D CNNs, various architectural designs, and often incorporate autoencoder structures. Such techniques have yielded diagnostic accuracies ranging from 84–95%, as reported in several studies [16–21]. Additionally, the MICCAI-BRATS (Brain Tumor Segmentation Challenge) annually conducts competitions for brain tumor segmentation, with outcomes and winners detailed on its website [22]. These challenges predominantly use MRI data and have led to significant developments, including a 3D DenseNet neural network aimed at determining the IDH genotype of gliomas, with an accuracy of 84.6% [23].

2D and 3D CNNs have been instrumental in classifying Alzheimer’s disease using MR imaging, as evidenced by various studies [24–26].

ISLES (Ischemic Stroke Lesion Segmentation) initiative regularly offers challenges aimed at the segmentation of ischemic brain lesions, predominantly utilizing MR imaging as noted in [27], with the latest challenges accessible on their website [28]. Current research on machine learning for diagnosing acute cerebral ischemic lesions in NCCT primarily focuses on techniques analyzing brain hemisphere symmetry [5, 29, 30], segmentation with generative networks in contrast-enhanced CT [31], and to a lesser extent, employs 2D [32] and 3D [2] CNNs. Regarding stroke analysis, several studies have implemented various artificial intelligence algorithms for the classification, segmentation, and diagnosis of strokes in NCCT and AngiographyCT images [5–7]. Mokli et al. [33] have conducted a review of the market’s available applications that utilize automatic and semi-automatic algorithms for image analysis in diagnosing acute cerebral infarction. Nevertheless, detailed information about the technical aspects of these algorithms, as well as specifics on the training and validation data, is often scant in the general descriptions provided on the applications’ websites [34].

### Goodness metrics

The metrics used to evaluate the performance of the networks were the *accuracy* and the *confusion matrix*. These are standard measures to assess the performance of any classifier, so they can be used to compare other alternatives and validate the efficiency of the solutions presented.

The accuracy definition is quite simple:

$$accuracy = \frac{true preditions}{total samples}$$

However, this metric alone does not allow for a more complete algorithm evaluation. A better way to evaluate the performance of a classifier is to use the confusion matrix. This matrix allows other concepts to be assessed, as will be seen below. To calculate the confusion matrix, a set of predictions is made with the network in question, with the test/evaluation set. Each row of the confusion matrix represents the actual class (positive/negative), and each column represents the class generated by the model (positive/negative). Some metrics from the confusion matrix can be used:

- True positives: the model predicts a value as true, and it really is true.
- True negatives: the model predicts a value as false, and it really is false.
- False positives: the model predicts a value as true, and it really is false.
- False negatives: the model predicts a value as false, and it really is true.

From these definitions, two additional metrics can be considered: precision and sensitivity.

$$precision = \frac{true \ positives}{true \ positives\ + \ false \ positives}$$

The sensitivity or true positive rate (recall) is the proportion of positive examples that are correctly detected by the classifier.

$$recall = \frac{true \ positives}{true \ positives\ + \ false \ negatives}$$

Depending on the problem being considered, it can be of interest to minimizing false positive cases, in which case precision could be used. On the other hand, sensitivity is the adequate metric if interest is focused on reducing false negative cases. In this specific case of stroke detection, it is very important to minimize false negatives which correspond to an incorrect stroke diagnosis. In other words, special attention to sensitivity must be considered. Unfortunately, improving on both metrics simultaneously is impossible, as increasing sensitivity reduces accuracy and vice-versa. Two other metrics from the confusion matrix can be deduced: *specificity* and the so-called *F1-score*, i.e., the weighted harmonic mean of accuracy and sensitivity.

$$specificity = \frac{true \ negatives}{true \ negatives\ + \ false \ positives}$$

$$F1-score = 2 * \frac{precision * recall}{precision + recall}$$

F1-score can be used as a valid reference metric as it weights the rest of the metrics. In this case, an excellent performance is considered when the value is 1 (or close to it) and a very poor performance when it is close to 0.

### Existing solutions

To validate the work proposal, it is also necessary not only to analyze the performance of the developed models but also the solutions that are presented in order to validate the results obtained. These solutions are presented here.

Presently, there are five leading software platforms that incorporate machine learning algorithms for stroke detection, including Brainomix e-ASPECTS (Oxford, UK), Olea Medical (La Ciotat, France), Siemens Frontier (Erlangen, Germany), iSchemaView RAPID (California, USA), and Viz.ai (California, USA) [35].

Viz.ai specifically utilizes a convolutional neural network for the analysis of head and neck CT angiography to detect occlusions in major vessels of the anterior circulation [36].

Both e-ASPECTS and RAPID ASPECTS platforms are clinically certified for stroke diagnosis and segmentation in NCCT, with e-ASPECTS boasting the most validation studies and comparability with expert radiologists’ performance [33]. As medical software, these platforms fall under the category of medical devices and are subject to validation and certification by regulatory bodies such as the US FDA (Food and Drug Administration) and the EU’s MDR (Medical Device Regulation).

Frontier ASPECTS is not yet certified, as documented [37]. Comparative studies reveal that while e-ASPECTS demonstrates high agreement with expert evaluations, Frontier’s performance aligns moderately [38].

E-ASPECTS and RAPID ASPECTS both endeavor to quantitatively evaluate focal ischemic damage utilizing the ASPECTS scoring system. They process complete cranial CT scans in DICOM format and use heat maps to demarcate affected areas. The distinction lies in their respective machine learning methodologies: Brainomix employs random forest learning for classification and segmentation, whereas RAPID ASPECTS involves skull and cerebrospinal fluid removal, uses atlases to identify the 20 ASPECTS score regions, and implements random forest learning for classification and segmentation.

These solutions require a previous segmentation in all cases to diagnose and classify ischemic stroke. This classification must be carried out in a single computer algorithm capable of being classified based only on NCCT images.

NCCT scans often suffer from low contrast and noise, making detection challenging. As in the case of previously presented solutions, some works focus on the segmentation of NCCT images, as presented in [39] and [40], with results similar to those achieved by neuroradiologists [41]. In fact, there are recent developments focused on using a combination of Transformers with CCNs [42]. Again, it focuses on automatic image segmentation but not image detection. A similar work is presented in [43] using a combination of NCCT and CTA (CT Angiography) images) to train a multi-scale 3D CNN. The work shows that if NCCT imaging alone is used, the accuracy achieved is about 53% (0.53±0.09), which is a very far result from what is expected (near 100%). Only by combining NCCT and CTA images accuracies of 90% (0.90±0.04) are achieved. [44] uses a combination of NCCT images with Computer Tomography Perfusion (CTP) images. A pair of NCCT-CTP images is used for each case studied. The proposed ensemble model leverages three deep Convolutional Neural Networks (CNNs) to handle three end-to-end feature maps and handcrafted features defined by distinct contra-lateral properties. The results obtained are promising, with 91.16% accuracy, but define three CCN networks’ complex structure. In the above works, a combination of different types of images is used, but none exclusively use NCCT images.

This research provides an initial requirement to use only NCCT images with signs of early ischemia that can be analyzed with neural networks, as segmentation techniques do with the ASPECTS score [45].

Under this assumption, other works use only NCCT images. In [46] a two-stage CCN model is trained, which effectively identifies and locates invisible ischemic strokes in non-contrast CT images. The model achieved up to 91.89% identification accuracy for ischemic stroke on NCCT images. In [47] another CCN is used which effectively detects and segments a specific type of thrombus (intra-arterial thrombi) using non-contrast computed tomography scans. The CCN has similar sensitivity and specificity to expert neuroradiologists (the paper compares CNN results with two radiologists’ decisions), achieving 0.86 sensitivity and 0.65 specificity in thrombus detection. CCN accuracy is not calculated, but it can be computed, resulting in a value of 85%, considering a prevalence size of 0.99 (very high value).

A more general ischemic stroke detector is presented in [48] The CNN model achieved a success rate of over 90% in distinguishing ischemic stroke cases from healthy controls in medical images. In all the papers presented, the accuracy is below 92%. This fact can define another research question: pre-trained networks can achieve better accuracy in detecting ischemic stroke by directly classifying NCCT images without previous segmentation.

In the case of the use of pre-trained architectures, architectures such as YOLO have been tested [49]. YOLO algorithm is used in object identification tasks. The AC-YOLOv5 algorithm, combining adaptive local region contrast enhancement and YOLOv5, effectively detects ischemic stroke in NCCt images with high accuracy and recall rates. Algorithm metrics have a high accuracy (91.7%) and recall (88.6%) rate. Again, accuracy metrics is under 92%.

Within the specific works using pre-trained networks, only this one has been found [50]. This research paper proposed a U-Net model based on the VGG-16 backbone, effectively detecting and segmenting infarct core areas in NCCT scans of ischemic stroke patients. Similarity indices such as the Dice coefficient and IoU are used as metrics and explicitly used in segmentation rather than classification. The U-Net model achieves an impressive Intersection over Union (IoU) score of 0.76 and a Dice coefficient of 0.79.

After analyzing the research in the area of ischemic stroke detection using NCCT images, it can be concluded that the use of pre-trained networks is not widespread and that the accuracies obtained by CNN networks do not exceed 92% in the case of non-pre-trained architectures. Therefore, this paper aims to show that it is possible to answer the research question formulated above: pre-trained networks can achieve better accuracy in detecting ischemic stroke by directly classifying NCCT images without previous segmentation.

An additional objective is to propose an Open CNN model that aids radiologists in decision-making, given the proprietary nature or non-utilization of neural network techniques in these applications’ algorithms. The attainment of this aim necessitates a specific Dataset, details of which will be delineated in the following section.

## Ictus (stroke) dataset

AI algorithms require high-quality, often pre-labeled data (with and without stroke, i.e., with and without hypodensities in the NCCT) to be effective. A critical preliminary step is to determine if there are specific datasets available for this research. A search for potential datasets in the area of stroke screening was conducted, and two possible sources were identified. The website [51] served as the primary source. This site hosts a vast collection of challenges in the realm of medical imaging. Two potential datasets were discovered. The first is related to the segmentation of cerebral infarction in CT angiography and magnetic resonance imaging, initiated by ISLES [28] in 2018. The second involves intracranial hemorrhage in non-contrast CT images from Kaggle [52], introduced in 2019. These datasets were unsuitable for evaluating early ischemia signs for several reasons. Some were CT scans with contrast (CT angiography), while others used different techniques, such as MRI. Additionally, some of the cases involved hemorrhagic strokes rather than ischemic strokes, which are the primary focus of this research. Consequently, it was decided to develop a specific dataset. To do so, understanding the structure and format of CT-acquired images is necessary.

### Computed tomography (CT) and hounsfiel units (HU)

In order to train neural networks, a dataset containing 3D images is required. These images are derived from cranial CT scans and involve the conversion of radiation intensity measurements from these scans (which can vary depending on the measuring equipment) into “Hounsfield” units or attenuation coefficient numbers. These units facilitate the creation of a density scale that is applicable to any image obtained with any equipment. To develop this scale, the attenuation produced by water under standard conditions of temperature and pressure on a ray beam was used as a reference, assigning it a value of zero (0 HU). The attenuation value of air under the same conditions is defined as −1000 HU, extending up to +1000 HU for dense and attenuated tissues.

Therefore, the Hounsfield scale has 2000 units with different shades, while the human eye cannot distinguish more than 40 and therefore cannot visualize much of the information. Only a partial scale of the HU values obtained in a CT study, a window, is represented to avoid this limitation. Using windows makes it possible to extract and display only part of the information obtained, which is interesting in each anatomical region, to visualize the organ or tissue to be studied.

**Fig. 2:**
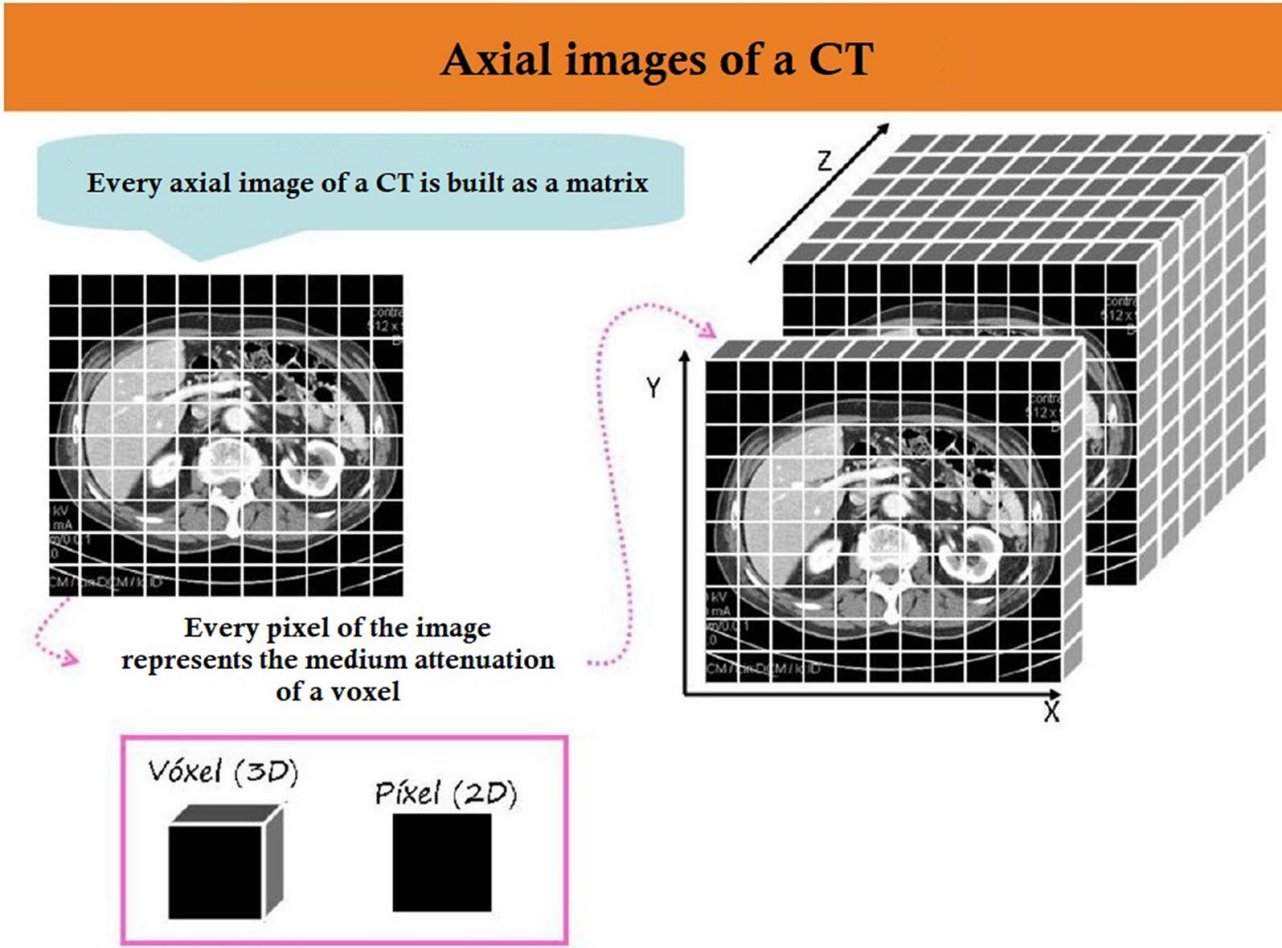
CT-image characteristics

These windows are defined by two parameters: their center or window level (WL), which corresponds to the middle grey of the visual scale and is assigned according to the density of the tissue to be evaluated, and their width (WW), which varies the range of greys on the visual scale. These parameters are different depending on the anatomical structures to be studied. Wide windows give less contrast. Narrow windows are used to study structures with close densities. The alteration in density that occurs in the early phase of infarction is usually very faint, which is why the radiologist improves visualization by reducing the window width to better distinguish diseased tissue from healthy tissue. For example, the center of the window is approximately −200UH (Hounsfield unit) and the window’s width is +1500. This window is called the pulmonary window. Other windows are the mediastinal window (WL=+50, WW=+350), the abdominal window (WL=+35, WW=+300), the bone window (WL=+300, WW=+1500) or the cerebral window, which is the one we use, which has WL=+40 and WW=+80, which allows us to differentiate the cerebral structures well thanks to the high contrast it provides. Thus, the anatomical structures will be represented by shades of grey, corresponding to their radiological density, measured in Hounsfield units. This enables the creation of both 3D images and 2D images (axial slices of the 3D image). The structure of the 3D image and the associated 2D images is illustrated in Fig. 2.

### Dataset structure

Acquiring a dataset of labeled cranial CT scans, both with and without stroke, has been a time-consuming and complex endeavor, necessitating several progressive steps. Image scans from various diagnostic modalities, such as tomography, ultrasound, magnetic resonance, and retinography, are typically in DICOM format [53]. This standard format for clinical image exchange and display includes not just the image but also personal details of the patient, their medical record number, and information about the equipment and location where the image was captured. In line with data protection laws, authorization was obtained from the leaders of the digital imaging department at the hospital for storing the CT studies in DICOM format. Once this consent was secured, the appropriate software was provided to facilitate the acquisition of CT analyzes in an anonymized manner.

#### Data collection

The non-contrast CT dataset in DICOM format comprises images from various hospitalized stroke patients between June 2015 and September 2020, during which the ictus code was activated. Studies indicating hemorrhage or other conditions not classifiable as ischemic stroke were excluded. These studies are categorized by two radiologists as normal (without hypodensities) or showing signs of ischemia (with hypodensity in one or more territories identified by the ASPECTS). The age of the patients and the degree of extension of the hypodensity are not considered, so if these parameters were taken into account, there could be differences in the results. The data selection, therefore, is sufficiently general to avoid possible biases, except for the patient’s sex (another possible feature that is not included). Of course, biases due to possible human error in the labeling of studies must always be taken into account.

**Fig. 3:**
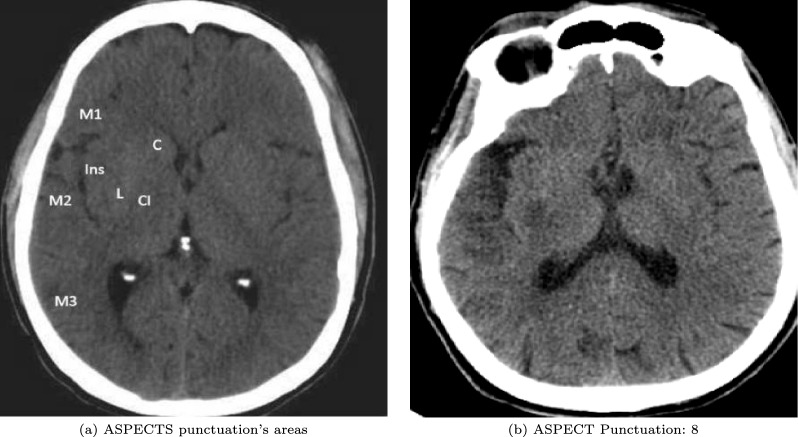
View of ASPECTS punctuation’s areas, detailing an ischemia in M2 and L areas

The data were divided into two groups: group 0, with stroke, and group 1, without stroke. All patients in group 0 exhibited some level of ischemic involvement in the MCA territory due to direct MCA obstruction by thrombus or embolus or secondary to carotid artery obstruction and showed hypodensity in CT according to ASPECTS criteria. The dataset records each patient’s identification, whether they belong to group 0 or 1, the affected regions, ASPECTS score (as shown in Fig. 3), and the affected cerebral hemisphere. Patients are clinically monitored, and complementary studies with cranial MRI are performed to confirm the accurate categorization of the tests in each cluster. Finally, the images are acquired in an anonymized DICOM format and securely stored in the cloud using Google Cloud Storage. The images are captured at a thickness of 3 mm using 64-slice multidetector CT equipment. Some older, isolated studies are captured using a helical CT with 5 mm slices.

**Fig. 4:**
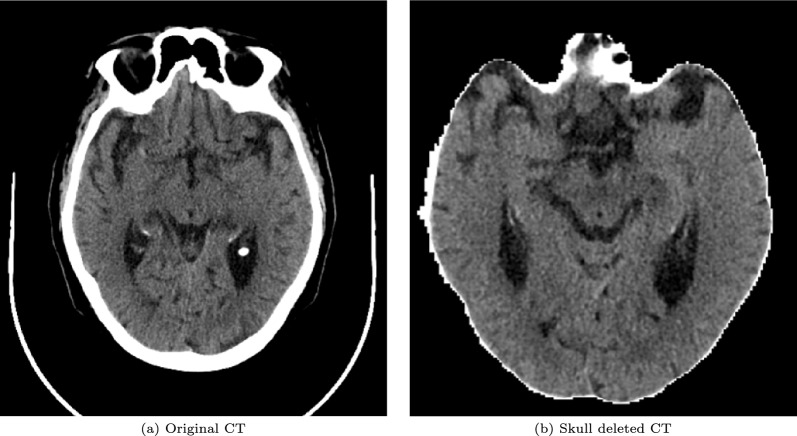
Comparison between original and skull deleted CTs

#### Processing data

The raw dataset is stored in DICOM format. Each CT study contains several axial slices of around 100, covering the whole brain volume with slices of 3 mm thickness. Data processing involves transforming this original data into matrices ready for the deep learning algorithm (python numpy format). Initially, the pixel values from the 2D image set contained in the DICOM format are read. As it was mentioned before, the numerical values in each pixel reflect the amount of energy absorbed by the tissues that the X-ray beam passes through. These values, together with the calibration values of the equipment (slope and intercept values), can be obtained from the DICOM file using the Pydicom library. This functionality allows the numerical values of each pixel to be transformed into Houndsfield units. Once we have obtained the 2D matrix of pixels of all the brain slices in HU, images are stacked to build the 3D matrix needed for the Deep Learning algorithms.

Two additional processing transformations have been used to improve the amount and quality of data. First, the bone was removed, and then data augmentation was applied. Data augmentation using rotations and translations of the original images has been performed considering the difficulty of obtaining many labeled medical images. Rotation (10 degrees to the right and 10 degrees to the left) and translation (10 pixels along the x-axis and 10 pixels along the y-axis) operations were performed to augment the data.

It was decided to remove the outline of the cranial bone to improve the quality of the images and eliminate elements not necessary for the detection of ischemia. It can be seen in the images that the intensity used by the skull (of a white tone) can interfere with the pattern recognition in the images, so it is necessary to try to reduce or eliminate this surface. There are different brain extraction algorithms such as *FSL-BET*, *3DSkullStrip*, *BrainSuite BSE*, *BEsST* or *ROBEX*.

For cranial bone removal, there is software for image segmentation such as *itk* or *3DSlicer* based on some of the algorithms described and others such as region growing, mask creation and other techniques. [54]. The *3DSlicer* software was used for the removal of the skull bone because of its free availability and ease of use. 3DSlicer provides the *SwissSkullStrip* algorithm [55], which removes cranial bone structures and eyes, and which we chose for two reasons: the resulting image maintains the DICOM format and does not modify the densities in the brain parenchyma image. The ResNet and DenseNet networks trained without bone removal failed to exceed an accuracy of 70–75% at best, respectively, rising to 75–80% after removal. Figure 4 shows an example of applying the deletion of the cranial bone.

Once the raw data is processed, a dataset of 264 stroke cases and 264 non-stroke cases has been created. Each of them is a 3D image, which can be divided into 2D axial slices. Each image has a sufficient diagnostic resolution (256 $\times$ 256 $\times$ 80 pixels).

Finally, since the number of images is not too large, classical data augmentation techniques (rotation, translation, among others) were implemented [56]. These operations resulted in a dataset with 1584 3D images covering the brain volume, 50% labeled with and 50% without stroke.

## Experimentation

The general methodology of the study follows a trial-and-error strategy based initially on parameters and hyperparameters from previous studies that have obtained good results in 2D image recognition. We then modify several key hyperparameters in our networks, one by one, selecting those that give us the best accuracy on the test data during training and discarding those with inferior results. First, we test the performance enhancements of our networks by increasing the batch size. Since these are networks with many parameters, they require significant processing power, and we have tested batch sizes up to the maximum that our resources allow. The literature suggests better results using mini-batches with sizes between 2 and 32 [57]. Subsequently, we assess the loss function and optimizers that work best with our data are evaluated, and finally, tests are carried out with different learning rates, both with and without reduction over time [58].

Different tests have been carried out to develop the experimentation, starting from simple architectures and gradually modifying these neural networks along with different parameters and hyper-parameters. A set of 3D deep convolutional neural networks based on well-known architectures with proven effectiveness in classifying 2D images from the Image-Net contest were implemented. The TensorFlow library was used for implementation, and the networks selected for their performance were VGG 3D, ResNet 3D, DenseNet 3D, and NasNet 3D. From now on, these architectures will be referred to by name only, omitting “3D” from the name but keeping in mind that they are networks for 3D image data.

### Non-pre-trained neural networks

The four networks mentioned above were tested as an initial step to verify that the implementation worked correctly with our data. The network architectures were used without loading the pre-trained networks’ hyperparameters (weights and bias) to evaluate their effectiveness and applicability in the stroke classification domain. All initial tests with the implemented network architectures yielded disappointing results. There was insufficient memory availability for VGG and NasNet due to their high number of hyperparameters. This limitation is critical for possibly incorporating the inference model into the radiologist’s decision support software. Thus, the DenseNet and ResNet networks were selected. However, issues such as overfitting and vanishing gradients in ResNet and DenseNet do not render them entirely useless as stroke classifiers based on untrained values. The initial parameters with which the first networks were trained are shown in Table 1.

**Table 1:** Training parameters for considered basic neural networks

Architecture	Optimizer	Learning rate (LR)	Initialization	Activation	Epoch	Batch
ResNet20	Adam	0.001	He-normal	ReLU	25	1
DenseNet121	Adam	0.001	Glorot	ReLU	25	2

An adaptive learning rate was used to enhance better performance, as this technique improves an increase in classification performance while reducing training time [59]. The initially selected loss function for ResNet was categorical *cross-entropy*, while binary *cross-entropy* was used for DenseNet. The accuracy obtained by these networks was relatively low and is shown in Table 2.

**Table 2:** Accuracy values for RestNet and DenseNet

Architecture	Training accuracy	Validation accuracy
ResNet	80%	66%
DenseNet	60%	55%

The results shown some improvement in the DenseNet network by changing the Adam optimizer to SGD, with a learning rate of 0.01, achieving training data accuracy of 0.79 and the test data of 0.6. However, neither network successfully converged to an acceptable solution in the test data.

Therefore, it was expected that results from non-pre-trained neural networks would lack statistical significance in terms of accuracy during training and validation/testing phases. Indeed, training with various proposed Convolutional Neural Network (CNN) structures produced inconsistent results, with accuracies falling below 60%.

As a result, the decision was made to utilize transfer learning techniques [60], specifically leveraging pre-trained networks known for their effectiveness in other domains of medical image detection, and particularly those utilizing 3D structures. The pre-trained CNNs chosen for this purpose were ResNet, DenseNet, and VGG. Interestingly, VGG, which had been previously disregarded, demonstrated significantly improved performance when implemented as a pre-trained network.

### Transfer learning techniques

Several versions were trained with different parameters across the three architectures, DenseNet 3D, ResNet 3D, and VGG 3D, using brain-extracted and data-augmented images, with 792 labeled with stroke and 792 without stroke.

To adapt the pre-trained 2D models from ImageNet to 3D medical imaging data, we extended the convolutional and pooling layers to three dimensions while maintaining the overall architectural structure. The pre-trained weights for ImageNet were used as initialization, specifically for the corresponding layers that could be seamlessly extended to 3D. Layers without a direct mapping from 2D to 3D were randomly initialized. Fine-tuning was then performed on the medical imaging dataset to optimize the weights for the specific task. This approach takes advantage of the feature extraction capabilities learned from natural images while allowing adaptation to volumetric brain data.

Regarding the dataset split, the data were randomly divided into two sets: 80% for training and 20% for testing. While we ensured an equal number of stroke and non-stroke cases in each set, we did not explicitly enforce stratification based on stroke subtypes.

#### DenseNet 3D

**Fig. 5:**
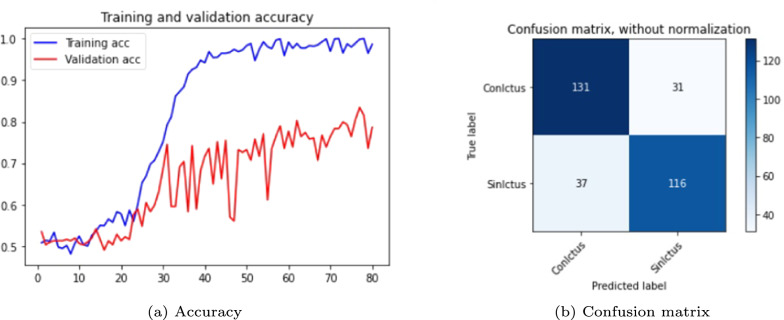
DenseNet results, version 1.1.1

**Fig. 6:**
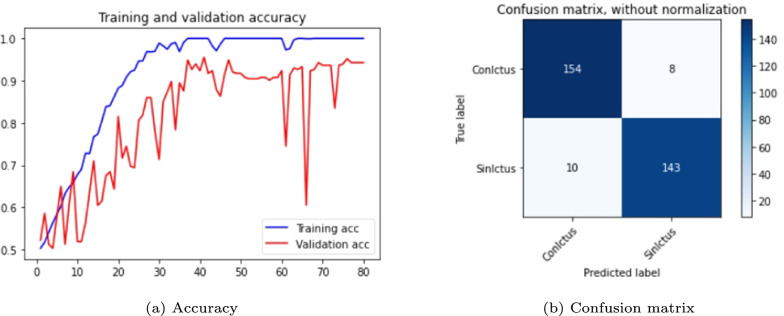
DenseNet results, version 1.2.1

**Fig. 7:**
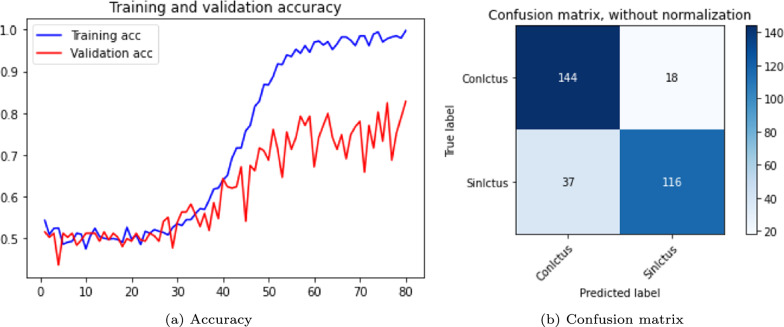
DenseNet results, version 1.3

The results are shown in the Table 3 and in the Figs. 5, 6 and 7. The figures display accuracy graphs for each epoch alongside the confusion matrices for the three versions that outperform their corresponding non-pre-trained network.

**Table 3:** DenseNet 3D results, batch size: 1–2

Version	Opt.	LR	Loss	Accuracy		Confusion matrix				Batch
				Train (%)	Test (%)	Sens.	Spec.	Prec.	F1-score	
1.0	SGD	0.001	Bin-ce	100	40	–	–	–	–	1
1.1	Adam	0.001	Bin-ce	100	50	–	–	–	–	1
1.1.1	Adam	0.001	Bin-ce	98	78	80%	75%	77%	0.78	2
1.2	SGD	0.001	Cat-ce	100	36	–	–	–	–	1
1.2.1	SGD	0.001	Cat-ce	100	94	95%	93%	93%	0.93	2
1.3	Adam	0.001	Cat-ce	99	82	88%	75%	79%	0.82	2
1.4	SGD*	0.001	Cat-ce	52	48	–	–	–	–	2

From Table 3, it is evident that with a batch size of 1, either with SGD or Adam optimizers; and loss function *binary* or *categorical cross-entropy*, the training results are suboptimal, exhibiting severe overfitting and test accuracy not exceeding 0.5.

The network shows significant improvement when the batch size to 2 (versions 1.1.1, 1.2.1 and 1.3). It is observed that the best results are obtained with a network with a SGD optimizer, using *momentum* of 0.9 and accelerated Nesterov gradient and loss function *categorical cross-entropy*. These parameters correspond to version 1.2.1 of the experiments with the DenseNet architecture.

A new version, 1.4, was developed with a DenseNet network with $lecun\_{normal}$ initialization, AlphaDropout and the SELU activation function. According to some authors [61], DenseNet is expected to perform better with these features. However, in this case, ir yielded poor results, failing to achieve high accuracy even on training data.

From the previous results, two key parameters were identified as critical for improving network performance: batch size (which should be increased) and learning rate (which should be reduced). Increasing the batch size demands higher computational power; therefore, cloud instances (Google Cloud Platform) with greater capacity were employed, with an additional cost. This decision enabled us to increase the batch size to 15 for DenseNet and 10 for ResNet. For these experiments, SGD was retained for the DenseNet and the Adam optimizer for ResNet, as they had demonstrated superior performance. The categorical *cross-entropy* loss function was also kept. Taking advantage of this computational upgrade, we reintroduced the VGG architecture into experiments, as it could new be reused. However, due to its high memory consumption, training was only feasible with a batch size of 2.

Another crucial parameter for optimizing neural network performance is the Learning Rate (LR). This parameter controls the magnitude of weight updates during training by determining the step size in the gradient computation for minimizing the loss function. If the learning rate is too high, the model may overshoot the minimum, preventing convergence or even causing divergence. In such cases, training is fast but unstable. Conversely, if the learning rate is too low, training becomes excessively slow and resource-intensive, making it necessary to find an optimal value or use adaptive mechanisms. Ultimately, there is no universally optimal learning rate, as its effectiveness depends on the specific dataset used for training.

In Table 4, several DenseNet networks were trained with a batch size of 15 (the maximum that our computational capacity allows), using the SGD optimizer. All versions, expect 2.0, incorporated an accelerated *Nesterov* gradient and employed the categorical *cross-entropy* loss function. The experiments analyzed the effects of learning rate changes at epochs 80 and 160.

**Table 4:** DenseNet 3D with batch size of 15 SGD optimizer and *categorical cross-entropy* loss function

Vers.	Epochs	LR	Accuracy		Confusion matrix			
			Train (%)	Test (%)	Sens. (%)	Spec. (%)	Prec. (%)	F1-score
2.0	80	0.01	95	80	64	96	95	0.76
2.1	80	0.001	97	87	77	98	96	0.85
2.2	160	0.001/0.0001 on epoch 80	98	95	95	96	96	0.95
2.3	160	0.001/0.0001 on epoch 30	96	91	90	92	92	0.90
2.4	160	0.001/0.0001 on epoch 20	97	92	93	91	91	0.91

**Fig. 8:**
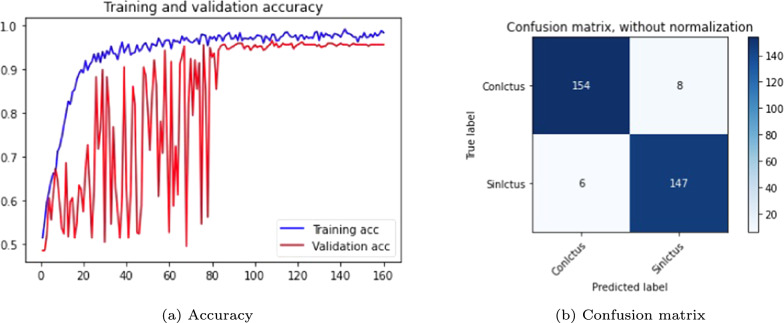
DenseNet 3D results, version 2.2

Version 2.2, which is shown in Fig. 8, achieves the best performance, demonstrating the highest sensitivity. It is important to highlight that this metric is particularly relevant, as the network must minimize false negatives to be reliable in the decision-making process. For clinical applicability, it is crucial that the model correctly identifies stroke patients and minimizes wrong classifications as normal cases.

#### ResNet 3D

**Fig. 9:**
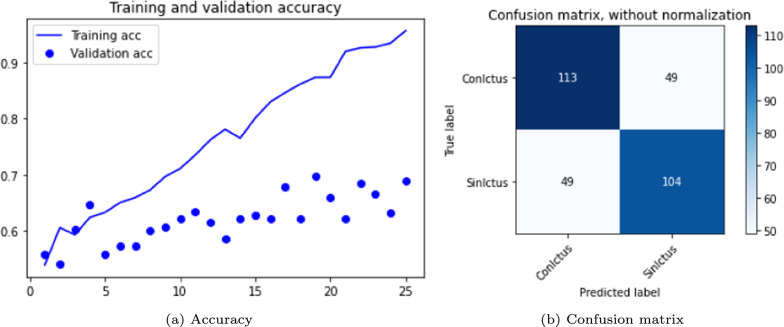
ResNet 3D results, version 1.0

**Fig. 10:**
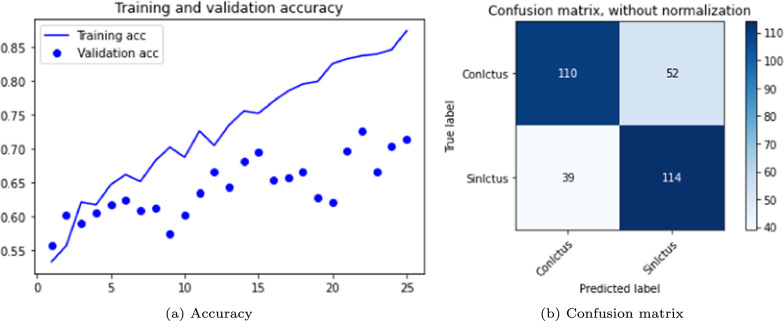
ResNet 3D results, version 1.1

Following the same procedure as in the DenseNet case, the experiments were performed with batch sizes of 1 and 2, incorporating the Adam optimizer. The same data was used, but the number of epochs was increased to 25. This type of neural network architecture requires significantly more time per epoch to process all samples compared to DenseNet. The results are shown in Table 5. Accuracy graphs and the confusion matrix are presented in Figs. 9 and 10.

**Table 5:** ResNet 3D results. Batch size 1 and 2

Version	Opt.	LR	Loss	Accuracy		Confusion matrix				Batch
				Train (%)	Test (%)	Sens. (%)	Spec. (%)	Prec. (%)	F1-score	
1.0	Adam	0.001	Cat-ce	95	68	69	68	69	0.68	1
1.1	Adam	0.001	Cat-ce	87	71	68	74	73	0.68	2

As with DenseNet, the following experiments focused on modifying the essential parameters, particularly the batch size. Table 6 summarizes the performance obtained with the ResNet versions with a batch size of 10 for 40 epochs with Adam and SGD optimizers. Training this network time-consuming, so only two two representative test versions were evaluated. Version 2.0, which employs the Adam optimizer, achieves higher accuracy in both training and test data, with reduced overfitting compared to version 2.1, which uses the SGD optimizer. However, the latter demonstrates higher sensitivity.

**Table 6:** ResNet 3D results. Optimizers: Adam and SGD. Loss function: *categorical cross-entropy*

Version	Epochs	LR	Accuracy		Confusion matrix			
			Train (%)	Test (%)	Sens. (%)	Spec. (%)	Prec. (%)	F1-score
2.0	40/Adam	0.001	95	79	75	83	82	0.78
2.1	40/SGD	0.001	100	76	77	77	78	0.77

**Fig. 11:**
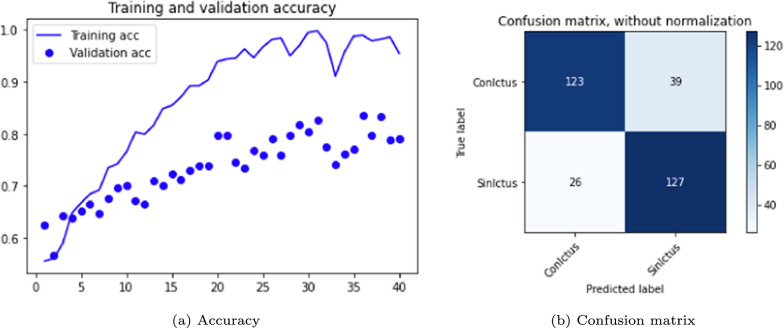
ResNet 3D results, version 2.0

**Fig. 12:**
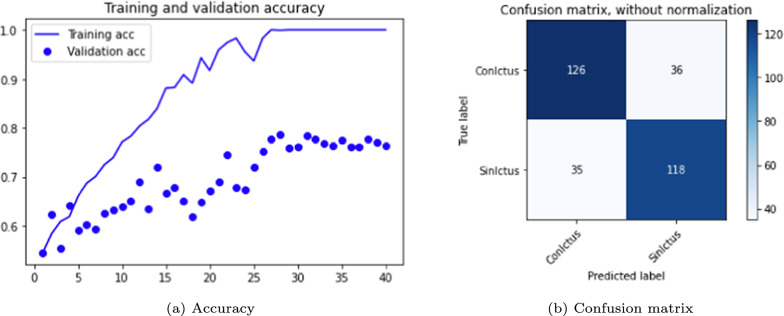
ResNet 3D results, version 2.1

ResNet also shows an improved performance with the increased batch size. The graphs and confusion matrices for both versions are shown in Figs. 11 and 12.

#### VGG 3D

In the case of the VGG 3D architecture, experiments have only been conducted with a small batch size computational limitation. It is important to note that the number of hyperparameters in VGG 3D is significantly higher than in DenseNet or ResNet, leading substantial RAM requirements for training and inference (179.1 million hyperparameters) [62].

**Fig. 13:**
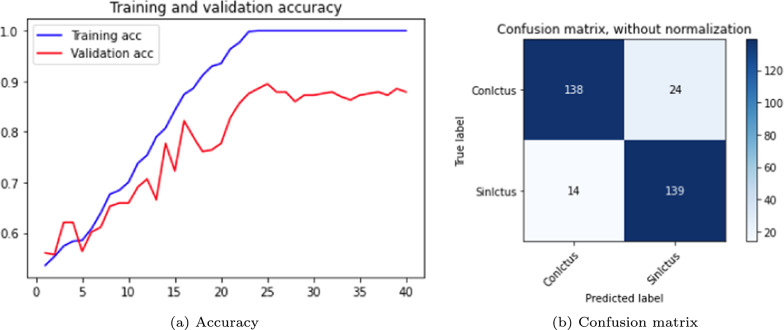
VGG 3D results: version 3.0

**Fig. 14:**
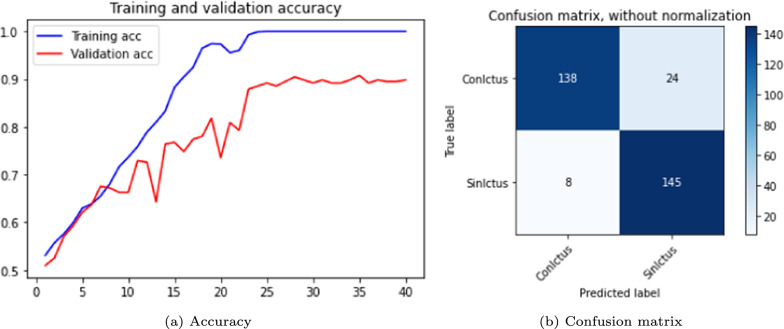
VGG 3D results, version 3.1

Table 7 shows the training results of the VGG network over 40 epochs, using the SGD optimizer and batch size of 2; the maximum that the computational capacity allows. The results indicated significant overfitting, though performance could likely improve with a larger batch size. Version 3.0 uses a constant LR of 0.001 throughout the 40 epochs, while version 3.1 starts with a LR of 0.001 until epoch 20, then decreases to 0.0001 until epoch 40, resulting in a slight improvement in test accuracy. Figures 13 and 14 show the corresponding accuracy plots and confusion matrices. Both versions outperform the ResNet network, but fall short compared to the DenseNet network.

**Table 7:** VGG 3D results. SGD and Adam optimizers and *categorical cross-entropy* loss function

Version	Epochs	LR	Accuracy		Confusion matrix			
			Train (%)	Test (%)	Sens. (%)	Spec. (%)	Prec. (%)	F1-score
3.0	40/SGD	0.001	100	87	85	90	90	0.86
3.1	40/SGD	0.001	100	89	85	94	94	0.88

## Results and discussion

Tables of results presented in the previous section allow us to identify the best version of the trained network for each neural network architecture used. For DenseNet 3D, an accuracy of 98% is obtained for the training data, while 95% for the accuracy with the test/evaluation data. A remarkable fact is that there is no network overfitting; therefore, the model could generalize the unseen cases well (images). It is also important to note that the sensitivity metric is high (95%) and allows the number of false negatives to be small. If the ResNet 3D architecture data is analyzed, the accuracy is at its best at 95% for the training data but rather worse for the evaluation/test data, which is 79%. Accuracy is generally worse than that obtained with the DenseNet 3D network.

The VGG 3D architecture produces excellent results for the training set, but there is a significant decrease in the case of the test/evaluation set. The training accuracy is 100%, and given the 89% accuracy of the best option (version 3.1), it can be indicated that there is over-fitting. This situation is more than likely due to the amount of image data available and could be verified by increasing this number. Given the work involved in labeling and generating the dataset, it is feasible for future work but not for this research. The existence of overfitting makes the model non-generalized and, therefore, not the most suitable model to be used in this particular case.

Regarding the convergence of the three models with the best metrics for DenseNet 3D, ResNet 3D and VGG 3D, it can be observed that DenseNet 3D has a better behavior as it converges faster. Regarding the accuracy metrics, VGG 3D has better results in the training set (100%) but has overfitting. In addition, the data for the sensitivity metrics are worse, as well as the accuracy in the test set.

A compromise between the different factors/metrics must be considered in determining the best solution. These are as follows:

- Accuracy of the neural network regarding the test/assessment set. This issue is critical as the model needs to be able to generalize to unseen stroke cases. This generalization is not feasible if the network accuracy metric for the training/test is 100% and there is overfitting.
- Sensibility of the neural network. It has been mentioned before that it is crucial not to diagnose stroke cases as if they were normal. This situation can lead to deaths and is a case that should be avoided at all costs (false positive).
- Neural Network complexity (number of hyperparameters). In order to implement the neural network inference as a stroke classifier, it is necessary to run this inference on a light computational system (a computer/personal computer without high performance). The fewer hyperparameters in the network, the lower the computational cost. In the VGG 3D case, the number of hyperparameters is high, while in DenseNet 3D or ResNet 3D is low (in comparison with VGG 3D).

Table 8 summarizes these factors for the best results on each neural network architecture. Given these factors or metrics, the best balance between them is achieved by the DenseNet 3D network. Therefore, the model chosen is DenseNet 2.2, with the parameters shown in Table 4.

**Table 8:** Factors considered for neural network 3D architecture selection

Factor/metric	Accuracy (%)	Sensibility (%)	Complexity
DenseNet	95	95	Low
ResNet	100	77	Low
VGG	100	85	High

## Conclusions and future works

This research exposes a trained binary classification of NCCT studies in cases with and without stroke as a main feature. No datasets are available for this particular case, so a new dataset has been built using real patient data. This dataset includes 3D images that train several CCN architectures from scratch, and later pre-trained networks. The research focuses on transfer learning techniques because experimentation with the more known 3D deep learning architectures has poor results in terms of metrics. Using pre-trained neural networks produces the best results, adapting the pre-trained hyperparameters to the particular case. Different parameters were tested to select the best neural network 3D architecture. For each network and parameter version, metrics were saved to compare these versions. DenseNet 3D network has excellent results regarding the factors chosen for the architecture being used as a possible assistant for decision-making. In addition to the metrics, the network complexity factor has been used to evaluate the architectures. This factor is crucial when implementing reference models to assist radiologists/physicians in detecting stroke. In clinical practice, the shorter the time from stroke to diagnosis and treatment, the better the prognosis. The availability of an artificial intelligence application that, during the NCCT scan, can rule out areas of hypodensity would assist the radiologist in deciding whether to extend the study and the neurologist in establishing treatment quickly. A significant research result is the building of the ictus dataset. This new dataset has been generated specifically for stroke detection, and there is no other known dataset, as the authors know. The number of cranial CT studies is sufficient to apply transfer learning techniques to perform the classification task. In addition, the DICOM format, a standard used worldwide for storing and communicating medical images, has made the construction procedure replicable and extensible to add new case studies and validate the neural network.

In future research, we aim to expand our dataset by incorporating a broader range of NCCT scans from diverse patient populations to enhance model robustness and reduce potential biases. Additionally, we plan to explore alternative deep learning architectures, particularly Vision Transformers (ViTs) and hybrid CNN-Transformer models, to evaluate their performance compared to traditional 3D CNNs. Another key area of improvement is the clinical validation of the model, where we intend to test our classifier with data from different hospitals and conduct comparative studies with radiologists to assess its real-world applicability. To facilitate its practical deployment, we will develop a graphical interface for easier interaction with medical professionals and work on optimizing the model for execution on standard clinical hardware. These improvements aim to make our AI-based stroke detection system more reliable, interpretable, and suitable for integration into existing diagnostic workflows.

## Data Availability

The images used in this research have been provided by the Hospital of Navarra and anonymized for research use. They are not publicly available, but can be obtained from the corresponding author, Rafael Pastor Vargas (rpastor@scc.uned.es), with the prior consent of the Hospital of Navarra.
